# Multi-stage gene normalization for full-text articles with context-based species filtering for dynamic dictionary entry selection

**DOI:** 10.1186/1471-2105-12-S8-S7

**Published:** 2011-10-03

**Authors:** Richard Tzong-Han Tsai, Po-Ting Lai

**Affiliations:** 1 Department of Computer Science and Engineering, Yuan Ze University, Chung Li, Taiwan, R.O.C

## Abstract

**Background:**

Gene normalization (GN) is the task of identifying the unique database IDs of genes and proteins in literature. The best-known public competition of GN systems is the GN task of the BioCreative challenge, which has been held four times since 2003. The last two BioCreatives, II.5 & III, had two significant differences from earlier tasks: firstly, they provided full-length articles in addition to abstracts; and secondly, they included multiple species without providing species ID information. Full papers introduce more complex targets for GN processing, while the inclusion of multiple species vastly increases the potential size of dictionaries needed for GN. BioCreative III GN uses Threshold Average Precision at a median of *k* errors per query (TAP-*k*), a new measure closely related to the well-known average precision, but also reflecting the reliability of the score provided by each GN system.

**Results:**

To use full-paper text, we employed a multi-stage GN algorithm and a ranking method which exploit information in different sections and parts of a paper. To handle the inclusion of multiple unknown species, we developed two context-based dynamic strategies to select dictionary entries related to the species that appear in the paper—section-wide and article-wide context. Our originally submitted BioCreative III system uses a static dictionary containing only the most common species entries. It already exceeds the BioCreative III average team performance by at least 24% in every evaluation. However, using our proposed dynamic dictionary strategies, we were able to further improve TAP-5, TAP-10, and TAP-20 by 16.47%, 13.57% and 6.01%, respectively in the Gold 50 test set. Our best dynamic strategy outperforms the best BioCreative III systems in TAP-10 on the Silver 50 test set and in TAP-5 on the Silver 507 set.

**Conclusions:**

Our experimental results demonstrate the superiority of our proposed dynamic dictionary selection strategies over our original static strategy and most BioCreative III participant systems. Section-wide dynamic strategy is preferred because it achieves very similar TAP-*k* scores to article-wide dynamic strategy but it is more efficient.

## Background

Gene normalization (GN) is the task of identifying the unique database IDs of genes and proteins found in literature. Even for trained biologists, GN is a difficult task that presents several problems making association with the correct ID number difficult. For one, gene and protein names often have several spelling variations or abbreviations. In other instances, gene products are described indirectly in a phrase, rather than being referred to by a specific name or code.

In many regards, the GN tasks of BioCreative II.5 & III are similar to those of previous BioCreative [1,2] workshops. However, they have two significant differences: firstly, they provide full-length articles in addition to abstracts; and secondly, instead of being human species-specific, they include multiple species and provide no species ID information. Both changes bring the BioCreative GN task closer to real-world curation of a model organism database.

The first difference, full-text articles, introduces more complex targets for GN processing. Unlike abstracts, full text articles contain many parts and sections, including the main freetext sections (introduction, methods, etc.), metadata, figure/table captions, notes, and so on. Each section or part has its own characteristics which we can use to guide GN and the ranking algorithm. For example, the Introduction section often contains information that repeatedly appears throughout the article (key genes), while the Results section presents new scientific findings, such as PPIs. Extracting a PPI from the Results section may require resolving an acronym whose full name has only been mentioned in the Introduction section. To exploit this type of section-specific information, we have developed a multi-stage memory-based GN procedure and a ranking method.

Predictably, the second difference, inclusion of multiple species, increases inter-species ambiguity. One gene name, abbreviation or code may refer to genes in multiple species, each with its own unique ID, or even to multiple genes in the same species or across different species. For example, without context, a search for ‘*tumor protein p53*, *TP53*’ in Entrez Gene may return results for proteins with the same name in over 20 species. Since the species in the context is unknown, all entries in the gene name dictionary must be loaded for GN. Currently, EntrezGene is the largest and most widely used publicly available gene or gene product database and has the best coverage of names and species. However if the billions of names that it contains are all loaded for GN, it greatly slows down the GN process.

Our GN system is designed to deal with the two changes above. To utilize the characteristics of different sections of a full-length paper, we use a three-stage GN procedure (see Methods section for details). In summary, the procedure is carried out starting from the sections with the richest context information (introduction) to those with the poorest. For our purposes, the informationally richest sections are those that are most likely to mention a gene’s full name [3]. Therefore, the introduction section is usually the richest section because it is here that authors first mention the genes of interest, giving their full names often followed by abbreviations used thereafter. The informationally poorest sections tend to be figure/table captions, which lack context information. Identifiers normalized in richer parts are used to help GN in poorer parts.

To handle the inclusion of multiple unknown species, we reduce ambiguity by dynamically selecting relevant entries from the dictionary for each paper or section and by employing an ID ranking model that sorts all genes in the paper according to confidence of correct normalization. By including species context features in the ranking model, we can improve inter-species accuracy. Many similar approaches have been proposed and proven effective [4,5]. BioCreative III gene normalization task data is used to evaluate our proposed strategies.

## Methods

**Figure 1:**
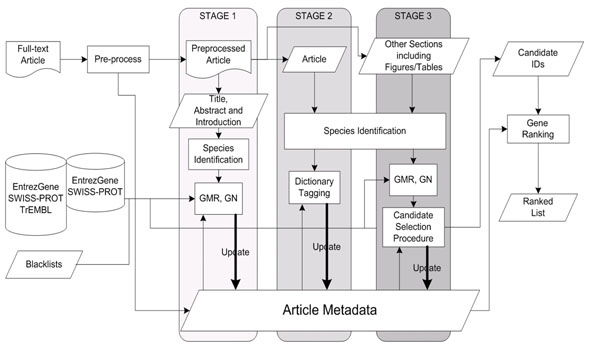
System workflow

Figure 1 shows a flowchart of our GN system. The well-formed full-text article is preprocessed to resolve the conjunction problems presented by Baumgartner et al. [6]. We use several rules proposed in [7] to expand collapsed ranges, such as "SOCS1-SOCS7", into their individual components "SOCS1, SOCS2, SOCS3, SOCS4, SOCS5, SOCS6 and SOCS7". In addition, preprocessing also generates article metadata as well as the full name/abbreviation mappings identified using Schwartz and Hearst’s algorithm [8].

After preprocessing, the multi-stage GN procedure is executed (Figure 1: stage 1 to 3). This method refines single-sentence-based GN by using section-specific information, scanning the whole article from the informationally richest to poorest sections—i.e. from the introduction section to table/figure captions.

The final step is ranking all normalized identifiers in a paper. We formulated the ranking problem as a support vector machine (SVM) classification problem, incorporating the confidence of the normalized identifiers and context information as features.

In the following sections, we explain the above steps in details and illustrate our strategies for selecting gene name dictionary entries for GN.

### Gene normalization

Three main subtasks are involved in our sentence-based GN method: gene mention recognition (GMR), dictionary matching, and disambiguation processing.

#### Gene mention recognition

The recognition of gene names is handled by a machine-learning (ML)-based gene mention tagger [9] trained on the BioCreative II gene mention dataset [10]. The GMR problem is formulated as a word-by-word sequence labeling task, where the assigned tags delimit the boundaries of any gene names. The underlying ML model is the conditional random fields [11] model with a set of features selected by a sequential forward search algorithm [12].

After GMR, we employ several post-processing rules developed in our previous work [7] to identify more gene mentions. For instance, if a parenthesized phrase follows an identified gene mention, we also regard the contents of the parentheses as a gene mention. The keywords, abbreviations, and full names recorded in the metadata are also used to adjust the gene mention boundary if the gene name string is a substring of them and vice versa. Take the sentence “Interaction between fortilin and **transforming growth factor-beta_GENE_** stimulated clone-22 (TSC-22) prevents apoptosis via the destabilization of TSC-22” as an example. The metadata stores the information that “transforming growth factor-beta stimulated clone-22” is the full name of “TSC-22”. Our GM tagger recognizes “transforming growth factor-beta” as a gene which is a substring of the full name stored in the metadata. As a result, the boundary is extended to include “stimulated clone-22”. The original string before adjustment is also stored in the metadata, which is checked when the adjusted gene name cannot be successfully mapped (in this example, the original string “transforming growth factor-beta” is also stored).

The recognized gene names are finally examined against a blacklist to filter out false positives. The list is automatically compiled from two databases, MeSH (for diseases), and HyperCLDB (for cell lines) [13], and the website NEB (for restriction enzymes) [14]. Our blacklist contains about 65,000 terms. When processing each article, our system dynamically updates the blacklist with synonyms (full names or abbreviations) according to the full-name/abbreviation mapping in the article metadata.

#### Dictionary matching

Dictionary-matching is able to assign candidate identifiers to each recognized gene mention. Two matching strategies are employed. The first uses a dictionary compiled by collecting gene names in EntrezGene and generating their orthographical variants[15]. Each recognized gene mention is looked up in the dictionary. If an exact match is found, then the gene is assigned that entry’s ID. Because all these terms are indexed by the Lucene search engine, we can then use the engine to find partial matches for each recognized gene mention.

If a gene mention is assigned two or more gene identifiers, we must determine which is more appropriate through disambiguation processing.

#### Disambiguation processing

The goal of disambiguation is to select the most likely gene identifier from multiple gene identifiers which share the same gene name. We manually constructed several rule-based classifiers which use context information, such as chromosome location, sequence length and so on, to determine the given identifier’s label. Each classification rule follows this general form:

*r*: (*Condition*) → *y* × *w*

The LHS of the rule (*Condition*) is a conjunction of attribute tests. The RHS is a value defined as *y* (1, 0 or -1), multiplied by *w*, a weight determined by proximity to the identifier mention (the same sentence: 1; the same section: 0.5). The final disambiguation process is based on the linear combination of the weighted scores of the various classifiers’ predictions. Some rules only have 1/0 values, such as chromosome location, because we have observed that this information may not always be described. Table 1 briefly summarizes the rules and classifiers. Take the rule, “Cell” (C), for example. For a given identifier *id*, the rule, C(*id*), checks the whole section in which *id* occurs for cell keywords (e.g. HELA, CHO, 3T3-L1). If it finds any matching keywords, C(*id*) returns 1 to indicate that there is a match. If keywords are found in other sections, C(*id*) returns 0. If no cell keywords for *id* are found in the entire article, C(*id*) returns -1.

**Table 1:** Rule-based classifiers

Species ^a^	S(*id*) refers to the species keywords of *id*
Cell ^b^	C(*id*) refers to the cell line keywords of *id*
PPI ^c^	PPI(*id*) refers to the interaction partner of *id*
History	
Full name/Acronym	FN(*id*) refers to the gene mention’s full name (its identifier is *id*)
Tissue ^c^	T(*id*) refers to the tissue keywords of *id*
Domain ^d^	D(*id*) refers to the domain keywords of *id*
Family ^d^	F(*id*) refers to the family keywords of *id*
MASS ^d^	M(*id*) refers to the MASS of *id*
Gene Ontology	GO(*id*) refers to the GO terms of *id*
Chromosome Location ^e^	CL(*id*) refers to the chromosome locations of *id*
Sequence Length ^d^	SL(*id*) refers to the sequence lengths of *id*
RS Number ^d^	R(*id*) refers to the RS number of *id*
The *id* refers to an identifier from the ambiguous list.	
The *nid* refers to a successfully normalized identifier stored in the metadata.	

^a^ Information collected from NCBI Taxonomy

*^b^ Information collected from Cell Bank*[23], *HyperCLDB*[24]* and Invitrogen*[25]

^c^*Information collected from Human Protein Reference Database* (*HPRD*)

^d^ Information collected from UniProt database

^e^ Information collected from EntrezGene database

### Multi-stage GN for exploiting the characteristic of different sections

Our three-stage GN procedure is shown in Figure 1.

#### Stage 1

In the first stage, GN is executed in the following order: Introduction, Abstract, Title. Successfully normalized identifiers are kept in memory (the metadata) for use in subsequent sections. We process the Introduction section first because the Abstract and Title sections are more concise and contain less contextual information and fewer identifiers. Following the order above, certain classifiers, including the PPI, Full-name/Acronym and the History classifier, are more effective. Take the PPI classifier for example. The classifier uses a gene's PPI information to disambiguate identifiers. As shown in Table 1, it requires a normalized identifier, *nid*, stored in the metadata. For each ambiguous gene identifier *id* the classifier checks whether *id* – *nid* is a PPI pair recorded in HPRD or not. If we process the article in a linear order (Title→Abstract →Introduction), the value of the PPI classifier will always be 0 when processing the Title (the same applies to the Full-name/Acronym and History classifiers). The values of other classifiers also tend to be 0 because of the lack of context information.

#### Stage 2

In this stage, the successfully normalized gene mentions and corresponding identifiers are extracted from the metadata to generate a dictionary. We then search the whole article for mentions in this dictionary. The Title, Abstract, and Introduction sections are also rechecked in case GMR missed any instances. When tagging gene mentions outside the Title, Abstract, and Introduction sections, the dictionary-based tagger also checks species keywords in the same sentence. If keywords are found and matched with the corresponding ID’s species, the ID is assigned. Otherwise, the tagger checks the metadata to see which species is the focus of the paper and assigns this to the mention. The focus species is determined by calculating the frequencies of the species keywords. The most frequent species is chosen as the focus species and is stored in the metadata.

Compared to directly employing a full list of gene names as a dictionary to annotate the whole article, this procedure can reduce the number of false positives [16]. It can also improve gene normalization accuracy in sections outside the Introduction section because an abbreviation’s full name can usually be found in the Introduction section.

#### Stage 3

**Figure 2:**
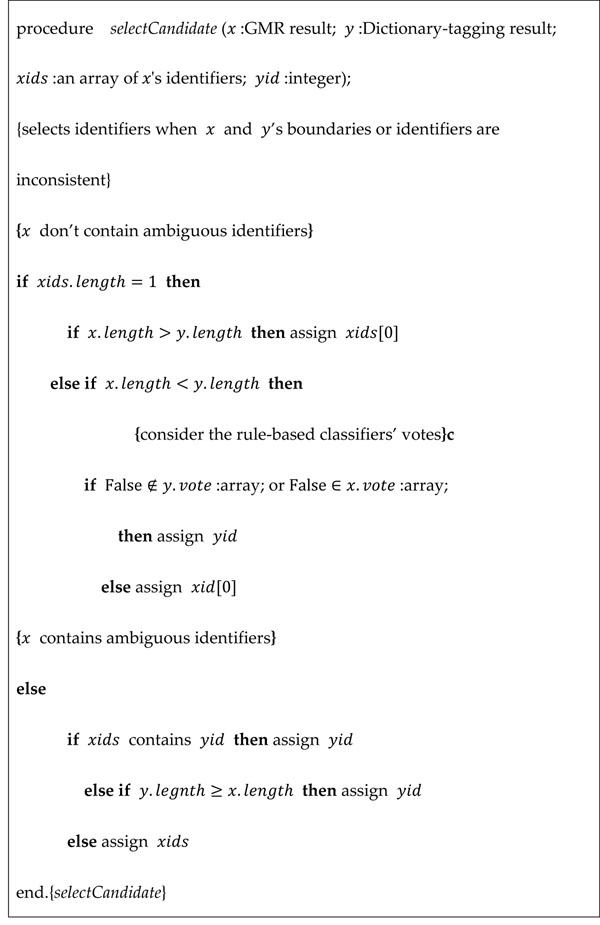
Candidate selection algorithm

The remaining paper sections (except Title, Abstract, and Introduction) including figure/table captions and appendix descriptions are processed by GMR+GN in the third stage. However, when GMR+GN is combined with the dictionary-based approach used in stage two, disagreement of boundaries or identifiers may occur. In each case, to select the most appropriate identifier, we designed a candidate selection algorithm, shown in Figure 2. This algorithm selects the ID with the longest gene mention string and the fewest rule-based classifier votes against it.

### Gene identifier ranking

In this stage, each normalized identifier from stage three is ranked by an SVM [17] classifier. For each identifier, the corresponding information stored in memory is used to extract features. In the following section, we describe the extracted features for gene identifier ranking.

#### GN matching method features

As mentioned before, there are two matching strategies to generate identifiers in our system: exact and partial matching. They are represented as Boolean features.

#### Disambiguation voting features

The value of the weighted vote generated by our disambiguation process is used as a feature. In addition, 13 Boolean features, which indicate whether or not the corresponding GN Classifier listed in Table 1 votes for the identifier, are also used as features.

#### Frequency features

The frequency with which the ID appears in the entire article is used as a feature. In addition, based on the work of McIntosh and Curran[18], who found that molecular interaction descriptions usually appear in the Results section, we added the percentage of an ID found in the Results section as a feature.

#### Location features

The locations where an identifier appears in the full text are extracted as Boolean features. Table 2 lists all locations which are taken into consideration. We also extract features for the last n sentences in the Abstract and Introduction because we have noticed that the key genes are often located at the end of those sections. This assumption is based on Swales’s Create A Research Space model [19] in which he shows that research articles contain three obligatory ‘moves’ in the Introduction section. He claims that most introductions end with Move 3 (occupying the niche) and should contain the announcement of principal outcomes.

**Table 2:** Location features

Location in full text article
Title
Abstract
Among the last *n*_1_^a^ sentences in the abstract
The first section (usually the introduction section)
Among the last *n*_2_^a^ sentences in the first section
The Results section
The other sections
The last section (usually the conclusion section)
Section, sub-section or paragraph titles
Appendix
Figure captions
Table captions

*^a^* In our configuration, n_1_ and n_2_ is set to 3 and 5, respectively.

#### Known information features

The information provided by the authors, including keywords and full-name/abbreviation definitions, is used to extract features. Table 3 shows the extracted feature sets.

**Table 3:** Known information feature sets.

Feature type	Description
Keyword match	A Boolean feature which indicates whether or not the identifier’s gene name matches keywords.
Full name/abbreviation match	A Boolean feature which indicates whether or not the identifier’s gene name matches full names or abbreviations.

### Strategies for reducing species complexity in gene name dictionaries

Most ambiguity in the GN process comes from the large number of existing gene names in dictionaries and the even larger number that results from the expansion of those original names. Inclusion of multiple species greatly compounds this complexity. Limiting gene dictionary size or excluding certain species’ genes may lessen the ambiguity and improve efficiency, but it may also lose crucial data. We propose two types of strategies for selecting relevant gene dictionary entries, static and dynamic.

#### Static strategy

Using a static strategy, the same set of terms is used in performing GN for every article. The sample static strategy that we designed for this paper uses only gene names from the 22 most common species in NCBI (from 7283 species).

#### Dynamic strategy

In the dynamic strategy, we use varying sets of names chosen according to the species context. The context can range from a sentence or paragraph to a whole section or even article, but in our system we only implement the latter two. We use two methods to detect the species in the context. The first is a keyword-based approach, which employs regular expressions to check for UniProt species keywords in the given section or article. If we identify keywords for certain species, we check only entries belonging to those species when performing GN.

## Results

### Dataset

BioCreative III participants were given a collection of training data that contains 32 full-text articles annotated by a group of experienced curators invited from various model organism databases. The articles are available in XML from selected journals in PubMed Central. A list of normalized EntrezGene IDs is provided for each article in the set.

The test data consists of 507 full-text articles. The organizers selected the 50 most difficult articles according to the results collected from the 14 participating teams and annotated these articles manually. They compiled these 50 articles into a test dataset (Gold 50). Furthermore, using the EM-algorithm-approach [20] they generated pooled results, which they compiled into a silver standard for all 507 test-set articles (Silver 507). They also compiled a silver standard 50 test set using the same 50 articles in the Gold 50 (Silver 50). Table 4 shows that there are many different species involved in this year’s GN task. We can see that the distribution of species among the three data sets is quite different (species in bold in the table are among UniProt’s top-22 most common species).

**Table 4:** Species distribution across data sets

#	Training Set (32 articles)	Test Set (50 articles)	Test Set (507 articles)
1	**S.cereviaiae (27%)**	**Enterobacter sp.638 (23%)**	**H.Sapiens (42%)**
2	**H.sapiens (20%)**	**M.musculus (14%)**	**M.musculus (24%)**
3	**M.musculus (12%)**	**H.Sapiens (11%)**	**D.melanogaster (6%)**
4	**D.melanogaster (10%)**	S.pneumoniae TIGR4 (9%)	**S.cerevisiae S228c (6%)**
5	**D.rerio (7%)**	S.scrofa (5%)	**Enterobacter sp.638 (4%)**
6	**A.thaliana (5%)**	M.oryzae 70-15 (4%)	**R.norvegicus (4%)**
7	**C.elegans (3%)**	**D.melanogaster (4%)**	**A.thaliana (2%)**
8	**x.laevis (3%)**	**R.norvegicus (3%)**	**C.elegans (2%)**
9	**R.norvegicus (2%)**	**S.cerevisiae S228c(2%)**	S.pneumoniae TIGR4 (2%)
10	G.gallus (2%)	E.histolytica HM-l (2%)	S.scrofa (1 %)
11	Other 18 species (9%)	Other 65 species (23%)	Other 91 species (7%)

### Evaluation metrics

**Figure 3:**
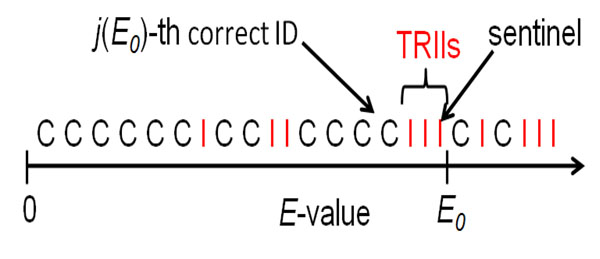
Example list returned by a GN system with correct (C) and incorrect (I) IDs illustrating the *j*(*E*_0_)-th correct ID, TPIIs and the sentinel ID

For an evaluation metric, BioCreative III uses ‘TAP-*k*’ (Threshold Average Precision at a median of *k* errors per query) [21], a measure closely related to the well-known average precision used in information retrieval, but also reflecting the usage of *E*-values in bioinformatics. The original E−value is a measure of the reliability of the S score. The S score is a measure of the similarity of the query to the sequence shown. In evaluating GN systems, the original TAP-*k* has been slightly modified. The E-value here measures the reliability of the score provided by each GN system. Let *E*_0_ be an arbitrary *E*-value threshold. For the query *q*, define *j*(*E*_0_) as the number of correct IDs in the list with an *E*-value less than or equal to the threshold *E*_0_. Consider the “terminal pre-threshold incorrect IDs” (TPIIs), the incorrect IDs retrieved after the *j*(*E*_0_)-th correct ID but having an *E*-value less than or equal to *E*_0_ (Figure 3). Call the last ID with an *E*-value less than or equal to *E*_0_ the ‘sentinel’ ID. Regardless of whether or not the sentinel is correct, it is associated with a precision *p*(*E*_0_), where *p*(*E*_0_) is the fraction of IDs preceding or including the sentinel that are correct. The following measure captures the effect of both post-threshold relevant records and TPIIs:(1)

To measure the overall retrieval efficacy for several sample queries, , the average of the TAP, , over all queries is adopted.

An *E*-value threshold *E*_0_ is determined to mirror a user's tolerance for errors. Assume that a user tolerates about *k* EPQ, *k* being some arbitrary integer. BioCreative III GN task gives *k* = 5, 10, 20 as an arbitrary but not unreasonable estimate of a tolerable EPQ. Determine the smallest *E*-value *E_k_*(*A*) corresponding to a median number of *k* EPQ over all queries *q* for a given system *A*. Thus, for any *E*-value threshold larger than *E_k_*(*A*), at least 50% of the queries have at least *k* errors. Each system's *E*-value predicts the actual number of EPQ with varying accuracy, so the threshold *E_k_*(*A*) depends on the algorithm *A*. With the same median *k*EPQ, all algorithms have the same specificity. With their specificities fixed at the same value, their sensitivities are on an equal footing, and therefore comparable. In summary, BioCreative III’s measure of overall GN efficacy is , the (query-averaged) TAP-*k* for a median *k* EPQ (the ‘TAP-*k*’), i.e. it is the average over all queries of Equation (1) with *E*_0_ = *E_k_*(*A*).

### BioCreative III results

Table 5 shows the results of our strategies and BioCreative iii’s average performance on the Gold-50 test set. Table 6 lists the results of our static strategy, dynamic strategy, and BioCreative III’s average performance on the Silver test sets. To show how each configuration’s performance relates to the individual performance of the BioCreative iii participating systems, we also append the results of top BioCreative III participating systems (see [21]) in the last three rows of Table 6. For each evaluation, top performance is bolded.

**Table 5:** Our strategies vs. BioCreative III participant average on gold-50 test set

Configuration	Test set gold standard 50								
	
	TAP5			TAP10			TAP20		
	
	TAP5	∆	relative improvement	TAP10	∆	relative improvement	TAP20	∆	relative improvement
BioCreative III Average (Baseline)	0.1421	-	-	0.1643	-	-	0.1764	-	-
Static strategy (Team 101_R3)	0.1773	+0.0352	+24.77%	0.2096	+0.0453	+27.57%	0.2374	+0.0610	+34.58%
Article-wide species	0.2012	+0.0591	+41.59%	0.2312	+0.0669	+40.72%	0.2480	+0.0716	+40.59%
Section-wide species	0.2007	+0.0586	+41.24%	0.2319	+0.0676	+41.14%	0.2480	+0.0716	+40.59%
Optimal Dynamic Dictionary	0.2708	+0.1287	+90.57%	0.3136	+0.1493	+90.87%	0.3140	+0.1376	+78.00%

**Table 6:** BioCreative III average vs. Static vs. Section-wide vs. BioCreative III top systems on silver test set

Configuration	Test set silver standard 50			Test set silver standard 507		
	TAP5	TAP10	TAP20	TAP5	TAP10	TAP20

BioCreative III Average (Baseline)	0.2175	0.2499	0.2690	0.2930	0.3062	0.3109
Static strategy (Team _101_R3)	0.3506 (+0.1331, +61.20%)	0.3942 (+0.1443, +57.74%)	0.3942 (+0.1252, +46.54%)	0.4351 (+0.1421, +48.50%)	0.4351 (+0.1289, +42.10%)	0.4351 (+0.1242, +39.95%)
Dynamic strategy: Section-wide species	0.3532 (+0.1357, +62.39%)	**0.4048** (+0.1549, +61.98%)	0.4024 (+0.1334, +49.59%)	**0.4951** (+0.2010, +68.98%)	0.4401 (+0.1339, +43.73%)	0.4401 (+0.1339, +43.73%)
Team_74_R3	**0.3747**	0.3747	0.3747	0.4555	0.4555	0.4555
Team_98_R3	0.3576	0.3953	**0.4499**	0.4086	0.4511	**0.4648**
Team_83_R1	0.3498	0.3531	0.3531	0.4581	**0.4581**	0.4581

In the first and the second rows of Table 5, we compare the scores of our static strategy, which uses only the most common species, to the average scores of the BioCreative III participants on the test set Gold 50. Our static strategy, which is our overall best performer on BioCreative III, exceeds the BioCreative III average by at least 24% in every evaluation. According to the BioCreative III GN task overview paper, our static strategy consistently remains in the top tier group in all evaluations [22].

The first and second rows of Table 6 show the results of the same configurations on the silver test set. Comparing the results with Table 5, we observe that its margins in the Gold 50 test set (24%-35%) are almost half of those in the Silver 507 test set (40%-50%). We believe this is because the majority of the most frequent species in the Silver 507 are among the 22 most common species in UniProt. On the other hand, only two of the top-10 species in the Gold 50 test set are among UniProt’s 22 most common species. This inspired us to try dynamic strategies to select relevant dictionary entries for context-specific normalization.

### Effects of dynamic strategies

Rows 3-5 of Table 5 shows the results of different strategies employed on the Gold 50. The first and second configurations (rows 3 and 4 of Table 5) dynamically enable dictionary entries based on whole-article or section context, respectively. Lastly, we show the best performance that could ideally be achieved by using a dynamic strategy with our GN system (row 5 of Table 5). We construct the ideal system as follows: For each article A, we find the species mentioned in A by checking each ID’s species in the gold standard ID list corresponding to A. For example, gene ID 10211 is found in article PMC2858709’s gold standard ID list. Gene ID 10211 belongs to Taxonomy ID 9606. Therefore, we know that this article mentions Taxonomy ID 9606.

As we can see in Table 5, both dynamic-strategy configurations increase all TAP-*k* scores by similar margins. In Tap-5, 10, and 20, they outperform the static baseline by about 17%, 13% and 6%, respectively. As *k* increases, the improvement margin of dynamic over static strategy decreases. This may indicate that more IDs can be correctly normalized in the beginning of the returned gene list after including the dictionary entries belonging to the context species in addition to the top-22 most common species. Take article PMC2887456 for example. Nad7 (ID:3800099) and EXPB11 (ID:778389) cannot be normalized because their species (Triticum aestivum, Taxnomy ID:4565) is not included in the top-22. However, using a dynamic strategy, the gene names corresponding to Triticum aestivum are included, and these two genes are correctly normalized and ranked as 3^rd^ and 8^th^. The TAP-5 score for this article is improved by 0.1944. The dynamic strategy can identify those gene IDs whose context information is rich but whose corresponding species is uncommon. If their dictionary entries are included, they can usually be correctly ranked in the front of the list, which affects Tap-5 more than Tap-10 or 20 and explains why as the *k* value increases, the advantage of a dynamic over a static strategy decreases.

As mentioned above, article-wide and section-wide contexts achieve very similar TAP-*k* scores. Consider the average normalization ambiguity in the test set: when using article-wide context, one gene name matches 2.5 IDs, while when using section-wide context, one gene name matches 1.7 IDs on average. When normalizing every occurrence of one gene in a given article using article-wide and section-wide contexts, 260,412 and 107,205 dictionary entries are enabled on average, respectively. Obviously, using section-wide context is more efficient.

Row 5 of Table 5 shows that the optimal dynamic strategy outperforms the proposed dynamic strategies by a significant margin. This implies that our GN system’s TAP score could be further improved with a better species identification system.

Employing the dynamic strategies on the silver test set also shows effectiveness. In Table 6, we can see that using the section-wide dynamic strategy, our GN system outperforms the best BioCreative III system in TAP-10 on the Silver 50 test set and in TAP-5 on the Silver 507 set. According to [22], using the silver standard allows GN developers to assess systems on the entire set of test articles without human annotation. This increases our confidence in the superiority of our proposed dynamic strategies over our original static strategy and most BioCreative III participating systems.

## Discussion

### No species keywords found

After analyzing our dynamic-strategy system’s results on the gold standard 50 dataset, we found that gene mentions belonging to rare species are often incorrectly associated with IDs belonging to popular species (such as human and rats). This is because our disambiguation process boosts the scores of IDs whose species information are found in the context. Since popular species’ keywords appear more frequently than those of rare species, IDs belonging to popular species are more likely to be selected.

### Distinct nomenclature of rare species

Another problem is caused by the inability of our GMR system to recognize genes belonging to rare species with distinct nomenclature (naming rules). We may be able to improve GMR in this regard by first generating pattern-based rules from names of more popular species using a local alignment algorithm such as Smith-Waterman.

## Conclusion

With recent advances in text-mining technology and increasing availability of full-text articles online, text mining can be carried out on full papers rather than just abstracts to expand and enrich automated literature curation. After an article has been selected for curation, a preliminary step is to list genes or proteins of interest in the article. While the concept is very simple, the task is very difficult to automate. In this paper, we present a multi-stage GN algorithm and SVM-based ranking method that we submitted to BioCreative III GN. We make use of the different characteristics of each paper section in our GN system. In addition, we propose two types of strategies for selecting dictionary entries for GN.

We have demonstrated that the static strategy that we submitted to BioCreative III, which uses only the most common species, exceeds the BioCreative III average by at least 24% in every evaluation. Examining this strategy’s much poorer performance in the Gold 50 test set, we noticed that most false negative IDs were of rare species. To improve identification of such species, we decided to try dynamic strategies to select relevant entries from the dictionary according to article-wide or section-wide species context. Our new approaches improved TAP-*k* scores by up to 17% in the Gold 50 test set. Our best dynamic strategy achieves comparable performance to the best BioCreative III systems in the silver-standard evaluation sets. These results demonstrate the superiority of our proposed dynamic strategies over our original static strategy and most BioCreative III participant systems. Section-wide dynamic strategy is preferred because it achieves very similar TAP-*k* scores to article-wide dynamic strategy but is more efficient. Comparison of our best results with an optimal configuration for which all species were verified manually shows that our GN system’s TAP score could be further improved with a better context-based species identification module.

## Competing interests

The authors declare that they have no competing interests.

## Authors' contributions

RTHT designed all the experiments and wrote most of this paper. PTL wrote the programs and conducted all experiments. RTHT guided the whole project.
